# A Pro-Inflammatory Biomarker-Profile Predicts Amputation-Free Survival in Patients with Severe Limb Ischemia

**DOI:** 10.1038/s41598-019-47217-1

**Published:** 2019-07-24

**Authors:** Hendrik Gremmels, Martin Teraa, Saskia C. A. de Jager, Gerard Pasterkamp, Gert J. de Borst, Marianne C. Verhaar

**Affiliations:** 1 Department of Nephrology and Hypertension, University Medical Center Utrecht, Utrecht, The Netherlands; 2 Department of Vascular Surgery, University Medical Center Utrecht, Utrecht, The Netherlands; 3 Department of Experimental Cardiology, University Medical Center Utrecht, Utrecht, The Netherlands; 4 Laboratory for clinical chemistry and haematology, University Medical Center Utrecht, Utrecht, The Netherlands

**Keywords:** Predictive markers, Peripheral vascular disease

## Abstract

Patients with Severe Limb Ischemia (SLI) have a high risk of amputation and mortality. Here, we investigated a panel of serum biomarkers with the aim of identifying biomarkers for major events and mechanisms that contribute to disease progression in established SLI. A panel of biomarkers including GROα, HGF, SCF, SCGFβ, SDF1α, TRAIL, IL-6, IL-8, FGFβ, GCSF, GMCSF, IP10, MCP1, PDGFbb, RANTES, TNFα, VEGF, sICAM, sVCAM, TM, and E-selectin was measured in serum samples from a subset (n = 108) of the JUVENTAS cohort. The primary outcome was major events, defined as major amputation or death. The inflammatory biomarkers IL-6, IL-8, GROα and IP-10 were significantly elevated in patients who reached a major endpoint. Results were validated in a secondary cohort (n = 146). Cox regression showed that adjusted hazard ratios were 1.40 (95% CI: 1.15–1.70, p = 0.0007) and 1.48 (95% CI 1.16–1.87, p = 0.001) for IL-6 and IP-10 in a fully adjusted model containing both biomarkers. A prediction model using IL-6 and IP-10 showed predictive accuracy with an AUC of ~ 78% in both discovery and validation cohorts, which is higher than previously published models. We conclude that inflammatory biomarkers predict major events in patients with SLI and allow the creation of biomarker-based risk-prediction models.

## Introduction

Peripheral Artery Disease (PAD) is one of the most prevalent manifestations of atherosclerosis, affecting over 27 million individuals in Europe and North America^1^. The most severe manifestation of PAD is termed Chronic Limb-Threatening Ischemia (CLTI) or Severe Limb Ischemia (SLI), which occurs when atherosclerotic lesions impede blood supply below the metabolic demands of the tissue even in rest. Patients with SLI present with symptoms of chronic rest pain and/or gangrene or ulcerations of the lower limb. SLI is associated with an unfavorable prognosis, with up to 20–40% of patients requiring a major amputation within one year of diagnosis^2^. The disease imposes a high socio-economic burden, particularly after amputation^3^.

Risk factors for the development of PAD and SLI show strong overlap with traditional cardiovascular risk factors and include age, smoking, hypertension, and diabetes^4,5^. The development of PAD is mainly caused by atherosclerosis of the lower limb arteries. The underlying pathogenesis is complex, involving an imbalanced lipid metabolism and a chronic inflammatory response in the arterial wall^6^. Genetic studies show that genes associated with inflammation, and endothelial remodeling are associated with the development PAD and SLI^7^, including IL-6, e-Selectin and Matrix Metalloproteases. Similarly, circulating biomarkers that have been associated with the development of PAD and SLI, including sICAM-1^8^, sVCAM-1^9^, CRP^10^ and IL-6^11^ reflect endothelial damage, oxidative stress, angiogenesis and inflammation^9,12^.

Relatively little is known about factors associated with disease progression and major clinical events in established SLI. Clinical signs and symptoms such as tissue loss and either excessively high or low Ankle-Brachial Perfusion Index (ABI) are associated with poor outcome^13^ as well as a history of cardiovascular events^14,15^.

In this study we aim to identify biomarkers that can be used to stratify risk and prognosis in SLI patients. We use baseline samples of no-option SLI patients included in the JUVENTAS cohort^16^ to identify predictors for major amputation or death. We have investigated a broad panel of biomarkers that reflect acute and chronic inflammation, endothelial damage and endothelial progenitor cell mobilization in a subset of the cohort. We investigate which of these processes is most closely associated with major outcomes and predicts events independently of established risk factors. The results are subsequently validated in the remaining JUVENTAS cohort as well as in an independent cohort of patients from the AtheroExpress study^17^ also including milder forms of PAD.

## Materials and Methods

### The JUVENTAS cohort and controls

The JUVENTAS study is a double-blind randomized placebo-controlled trial investigating bone marrow-derived mononuclear cell (BM-MNC) therapy for no-option SLI; details of the trial design^18^ and results^16^ have been described elsewhere. Patient inclusion was open from 2006 until 2012.

Inclusion criteria were severe infra-popliteal PAD, defined as severe intermittent claudication (Fontaine IIB), ischemic rest pain (Fontaine III) or non-healing ischemic ulcers (Fontaine IV) and ineligibility for angioplasty or bypass surgery, as well as an Ankle-Brachial Index (ABI) ≤ 0.6 or unreliable measurement.

After inclusion, patients underwent bone marrow aspiration and were randomized to receive either 3 intra-arterial injections of autologous BM-MNCs at inclusion, week 2 and week 6 or matching placebo injections. Death and major amputation, defined as amputation through or above the ankle joint, at 1 year after inclusion were recorded as primary endpoints. Amputation-free survival (AFS) was used as combined endpoint. For the present report the study report was amended to extend follow-up until December 2014. Healthy control subjects (n = 34) of similar age (median age 65 years) and gender were recruited from hospital personnel.

The institutional review board of the University Medical Center Utrecht approved the study protocol (METC # 06-030/O). The study was conducted according to the Declaration of Helsinki, and all patients provided written informed consent prior to the study interventions.

The first consecutive 108 patients of the JUVENTAS cohort were designated as discovery cohort. The complete panel of biomarkers was investigated in these patients, as well as in the age-matched control population.

### AtheroExpress

The AtheroExpress biobank is a prospective biobank study that includes specimens from patients undergoing carotid or iliofemoral endarterectomy^17^. Follow-up data during a 3-year period were obtained through questionnaires sent to patients and cardiovascular events were validated using health records kept by general practitioners. For the purpose of this study, 66 patients with PAD (Fontaine grade II-IV) were randomly selected of whom complete follow-up was reviewed and available cryopreserved serum used.

For the validation cohort, the 52 remaining patients in JUVENTAS and the 66 AtheroExpress patients were pooled into a single cohort. Only the variables available in both cohorts are reported here.

### Multiplex analysis and quantification of cytokines, chemokines and CAMs

Biochemical parameters (i.a. liver enzymes, kidney function, lipid spectrum, glucose and homocysteine level) and complete cell counts were measured using standard clinical laboratory procedures. A multiple cytokine assay (Bio-rad, Hercules, CA) was used to determine a panel of cytokines and growth factors consisting of basic Fibroblastic Growth Factor (bFGF), Granulocyte-colony stimulating factor (G-CSF), Growth regulated oncogene-alpha (GRO-α), Hepatocyte Growth Factor (HGF), Interleukin-6 (IL-6), Interleukin-8 (IL-8), Interferon gamma-Induced Protein 10 (IP-10), Monocyte Chemotactic Protein 1 (MCP-1), Platelet-Derived Growth Factor-bb (PDGF-bb), Regulated upon Activation Normal T-cell Expressed, and presumably Secreted (RANTES), Stem Cell Factor (SCF), Stem Cell Growth Factor-beta (SCGF-b), Tumor Necrosis Factor-alpha (TNF- α), Tumor Necrosis factor related Apoptosis Inducing Ligand (TRAIL), and Vascular Endothelial Growth Factor (VEGF), soluble E-selectin (sE-selectin), Thrombomodulin (TM), soluble Intercellular Cell-Adhesion Molecule 1 (sICAM) soluble Vascular Cell Adhesion Molecule 1 (sVCAM) and Stromal Derived Factor 1 alpha (SDF1a). Assays were performed using Bio-plex protein array with manufacturer-supplied software as in^19^. Values below the detection range were set to the lower limit of quantification.

### Statistical analysis

Statistical analyses were performed using the ‘R’ statistical programming environment. Continuous data are reported as mean ± standard deviation (S.D.) when normally distributed, or as median and interquartile range [I.Q.R.] when skewed, unless otherwise indicated. An independent samples Student’s t-test was performed for normally distributed data, a Mann-Whitney-U test for non-normally distributed continuous data and a Fisher’s exact test for categorical data. Time-to-event analysis was performed by the Kaplan Meier method or Cox regression. In analyses within the JUVENTAS cohort, the effect of treatment arm was examined and stratified for if necessary. For biomarkers the Hazard Ratios (HR) are given for log-transformed values, thus indicating and e^HR^ increased risk. The proportional hazards assumption was checked for all models by visually examining Schoenfeld residuals. Prediction models were made using multivariable logistic regression; different models were compared on Akaike Information Criterion (AIC), using forward- and backward model factor inclusion. To correct for over-fitting in logistic regression models, global shrinkage of parameters was applied using the ‘jack-knife’ method (as applied in the R package ‘shrink’). ROC-curves are shown with confidence bands obtained by resampling with replacement (‘bootstrapping’) in 2000 iterations, the R package ‘pROC’ was used. P-values < 0.05 were considered to be statistically significant.

## Results

### Baseline characteristics and follow-up of discovery cohort

For the purpose of identifying biomarkers associated with clinical outcomes we split the JUVENTAS cohort and included the first 108 consecutive patients (67.5%) in the discovery subset. Patients were followed for a median of 5.6 years in the discovery cohort, during which time 34 amputations and 32 deaths occurred, leading to 53 major events for primary analysis.

Median Age was 66 years, the majority of the patients was male and had a high burden of concomitant cardiovascular disease. Patients who suffered amputation or death were more likely to be older and had more advanced PAD (see Table 1).

**Table 1:** Baseline characteristics of the Discovery Cohort and Controls.

Juventas Discovery Cohort	Total Cohort (n = 108)	Primary Endpoint (n = 53)	No Primary Endpoint (n = 55)	P-Value	Control
Sex (M/F)	74/34	42/9	32/23	**0**.**03**	21/13
Age (yrs)	66 [58–74]	70 [61–76]	62 [54–72]	**0**.**009**	65 [60–72]
BMI (kg/m2)	26.7 (4.6)	26.7 (4.9)	26.7 (4.31)	0.97	23.2 (2.32)
Smoking (Current/Past/Never)	26/69/13	12/33/8	14/36/5	0.63	0/7/27
Systolic BP (mm Hg)	129.91 (19.7)	130.3 (21.3)	129.5 (18.2)	0.83	128 (23.3)
Diastolic BP (mm Hg)	72.7 (9.7)	72 (10.4)	73.4 (9.0)	0.44	72.5 (8.8)
Urea (mmol/l)	6.0 [4.1–8.3]	6.9 [4.0–11.6]	5.6 [4.2–6.8]	0.09	—
Creatinine (umol/l)	91 [76–112]	102 [76–144]	88 [77–108]	0.19	80 [76–90]
GFR (MDRD, ml/min/1.73 cm2)	69 (27)	68.4 (32.2)	69.9 [22.1]	0.78	78 [72–87]
Cholesterol (mmol/l)	4.30 (1.11)	4.12 (1.05)	4.48 (1.15)	0.09	4.91 (0.96)
HDL (mmol/l)	1.21 (0.45)	1.13 [0.48)	1.29 (0.40)	0.06	1.41 (0.53)
Triglycerides (mmol/l)	1.45 [0.9–2.0]	1.4 [1.0–1.9]	1.5 [0.8–2.0]	0.57	0.6 [0.6–0.8]
Hemoglobin (mmol/l)	8.16 (1.09)	7.9 (1.0)	8.5 (1.1)	**0**.**006**	8.9 (0.81)
CRP (mg/ml)	5.34 [2.1–11.9]	6.4 [2.8–13.3]	4.13 [1.7–11.1]	0.13	—
History of CVA	8 (7.4%)	8 (15.1%)	0 (0%)	**0**.**009**	0 (0%)
History of MI or Angina	42 (38.9%)	26 (49%)	12 (22%)	**0**.**012**	0 (0%)
History of Major Amputation	9 (8.3%)	5 (9.4%)	4 (7.3%)	0.95	0 (0%)
History of Dialysis	3 (2.8%)	1 (1.9%)	2 (3.6%)	0.99	0 (0%)
Diabetes (No, NIDDM, IDDM)	66/20/22	28/13/12	38/7/10	0.18	34/0/0
ABI	0.53 (0.31)	0.42 [0.2–0.6]	0.54 [0.5–0.7]	**0**.**021**	—
Rutherford class (3/4/5/6)	7/36/61/4	0/14/36/3	7/22/25/1	**0**.**008**	0
Fontaine class (IIB,III,IV)	7/36/65	0/14/39	7/22/26	**0**.**003**	0
Ulcer	65 (60.0%)	39 (74%)	26 (47%)	**0**.**009**	0 (0%)
Ulcer Area (cm2)	1.73 [1.0–4.3]	2.38 [1.0–5.1]	1.35 [0.8–2.4]	0.06	0
BMMNC Treatment (No/Yes)	53/55	27/26	28/27	0.99	0 (0%)
Anti-Platelet Drugs	74 (69%)	37 (70%)	37 (67%)	0.32	0 (0%)
ACEi or ARB	62 (57%)	33 (63%)	29 (53%)	0.41	0 (0%)
Diuretic	53 (49%)	29 (55%)	24 (44%)	0.34	0 (0%)
Beta blocker	48 (44%)	28 (53%)	20 (36%)	0.13	0 (0%)

Values in square brackets indicate interquartile range [IQR], values between regular brackets indicate standard deviation (SD) or percentage, where indicated. The p-value reflects the comparison between event vs no event groups.

### Differences in biomarker abundance

Levels of GROα, HGF, SCF, IL-6, IL-8, G-CSF, IP-10, VEGF, sICAM-1, sVCAM-1 and TM were higher in SLI patients compared to controls (see Supplementary Fig. 1).

**Figure 1:**
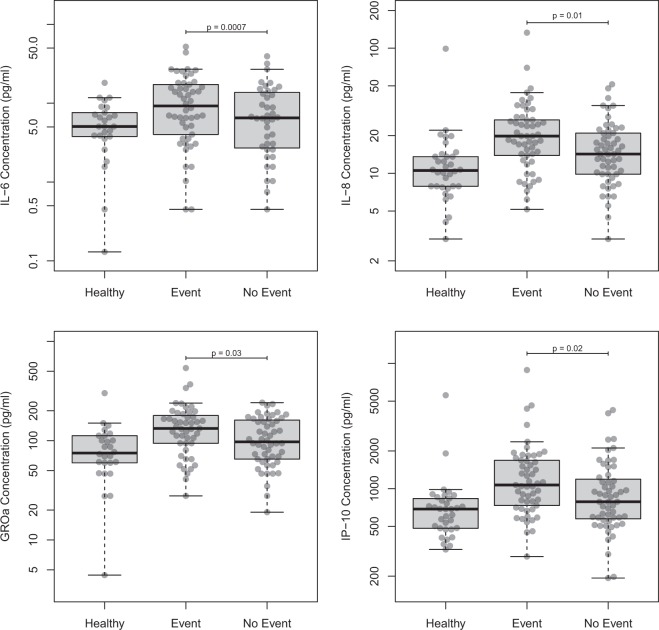
Cytokine Measurements. Boxplots showing levels of biomarkers that discriminated between patients with and without major outcome. Healthy Controls are shown as reference Box indicates median and interquartile range, whiskers indicate range.

Only GROα, IL-6, IL-8 and IP-10 were able to discriminate between patients who were to experience an event, compared to patients who remained event-free (Fig. 1). Average values in no-events versus events were: 96.8 (83.1–112.7) versus 125.2 (106.6–147.0) pg/ml for GROα (p = 0.03), 5.8 (4.3–7.6) versus 8.0 (6.0–10.7) pg/ml for IL-6 (p = 0.0007), 14.4 (12.3–19.3) versus 19.4 (16.4–22.9) pg/ml for IL-8 (p = 0.01) and 851.4 (719.5–1007.5) versus 1150.8 (970.3–1364.9) pg/ml for IP-10 (p = 0.02, all data are geometric means ± 95% CI).

### Inflammatory biomarkers are associated with amputation-free survival

We next investigated whether the four biomarkers proved independent predictors for major endpoints in time-to-event analysis by creating Cox regression models. In univariate analysis all four biomarkers proved significant predictors of major events. HRs were 1.76 (95% CI: 1.0–3.0, p = 0.04) for GROα, 1.58 (95% CI: 1.24–2.0, p = 0.0002) for IL-6, 1.93 (95% CI: 1.3–2.9, p = 0.002) for IL-8, and 1.62 (95% CI: 1.1–2.4, p = 0.01) for IP-10. Analysis of separate endpoints showed that IL-6, IL-8 and GROa more closely predicted amputation, rather than mortality, whereas for IP-10 there was no difference (see Supplementary Table 2 for the full data).

**Figure 2:**
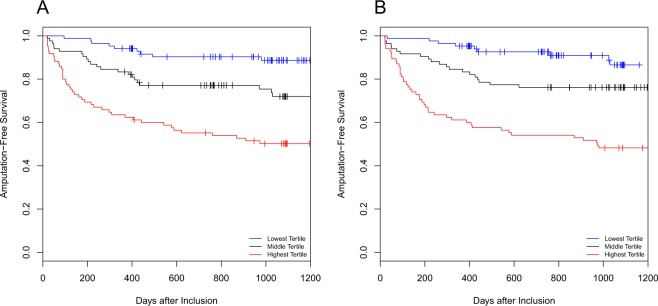
Survival Curves. Patients were divided in tertiles based on plasma levels of biomarkers, panel A shows the curves for IL-6 and panel B for IP-10.

When we examined combinations of biomarkers in multivariable analysis, we observed a high degree of correlation between IL-6, IL-8 and GROα (Supplementary Fig. 2). IL-6 and IP-10 proved the best combination to predict AFS, with adjusted HRs of 1.52 (1.2–1.9) and 1.45 (0.98–2.15, Fig. 2).

### Validation and adjustment for confounding

We proceeded by validating the two best biomarkers, IL-6 and IP-10 in a validation cohort comprised of the remaining JUVENTAS cohort combined with an independent selection of patients drawn from the AtheroExpress registry study. Characteristics of the validation cohort are presented in Table 2.

**Table 2:** Baseline characteristics of the Validation Cohort.

	No Primary Endpoint	Primary Endpoint	p-value
	n = 120	n = 26	
Sex (Male)	88 (73.3%)	19 (73.1%)	0.999
Age (yrs)	69 [61, 76]	73.00 [69, 80]	**0**.**037**
BMI (kg/m2)	26.37 (3.60)	26.61 (4.71)	0.777
Smoking (% Current)	35 (29.4)	5 (19.2)	0.418
Systolic BP (mm Hg)	140.10 (22.44)	145.90 (25.04)	0.260
Diastolic BP (mm Hg)	75.76 (11.89)	71.90 (11.93)	0.150
Creatinine (umol/l)	83.00 [68.00, 100.00]	115.00 [67.00, 139.00]	**0**.**010**
eGFR (MDRD, ml/min/1.73 cm2)	82.01 (24.80)	65.42 (31.88)	**0**.**005**
Cholesterol (mmol/l)	4.45 (1.13)	4.22 (1.52)	0.502
HDL (mmol/l)	1.20 (0.38)	1.05 (0.29)	0.139
Triglycerides (mmol/l)	1.65 [1.20, 2.35]	1.70 [0.80, 2.20]	0.428
Hemoglobin (mmol/l)	8.47 (0.99)	7.94 (1.29)	**0**.**020**
CRP (mg/l)	3.69 [1.37, 11.73]	13.68 [3.31, 126.06]	**0**.**014**
No history of Dialysis	119 (99.2%)	25 (96.2%)	0.789
History of CAD	68 (56.7%)	12 (48.0%)	0.568
History of CVA	96 (91.4%)	19 (79.2%)	0.168
History of Diabetes	42 (35.0%)	7 (26.9%)	0.574
ABI	0.58 [0.45, 0.70]	0.52 [0.45, 0.62]	0.437
Fontaine Class			0.190
Fontaine II	37 (36.6%)	5 (21.7%)	
Fontaine III	31 (30.7%)	6 (26.1%)	
Fontaine IV	33 (32.7%)	12 (52.2%)	
Anti-platelet Medication	99 (82.5%)	23 (88.5%)	0.651
Beta-Blocker	56 (46.7%)	16 (61.5%)	0.247
Diuretic	50 (41.7%)	15 (57.7%)	0.203

Values in square brackets indicate interquartile range [IQR], values between regular brackets indicate standard deviation (SD) or percentage, where indicated. The p-value reflects the comparison between event vs no event groups.

Both IL-6 and IP-10 levels were higher in patients that reached a major endpoint, compared to patients who did not (2.86 pg/ml [1.8–4.4] and 190.5 [127–285] pg/ml versus 1.26 pg/ml [1.0–1.5] and 90.6 pg/ml [76–108]). Unadjusted and mutually adjusted HRs for IL-6 and IP-10 were roughly similar in the validation cohort compared to the discovery cohort (1.60 [1.2–2.1], p = 0.0007 vs 1.43 [1.1–1.9], p = 0.01 for IL-6, and 1.77 [1.2–2.5] p = 0.001 vs 1.57 [1.1–2.3], p = 0.01 for IP-10 respectively). In order to retain sufficient power to allow for full adjustment we decided to pool discovery and validation cohorts in multivariable analysis.

In adjustment for sex, we observed that the proportional hazards assumption was not met, i.e. that men and women appeared to have different (and crossing) survival curves. We therefore stratified the analysis by gender in multivariate models, in order to maintain validity of the models. We did not observe a difference between IL-6 and IP-10 levels in men and women, however, nor was there an interaction with sex in Cox regression.

HRs remained virtually unchanged after adjustment for age and sex (Table 3, Model 2) and only minimally changed after full adjustment for Age, Sex, GFR, Presence of Diabetes, Hb, ABI, and Fontaine Classification (Model 3). Further inclusion of C-reactive Protein (CRP) in the model showed that IL-6 and IP-10 predict major endpoints largely independently of CRP (Model 4).

**Table 3:** Cox Regression results in the Combined Cohort. Model 1 shows mutually adjusted HRs for IL-6 and IP-10.

	HR IL-6	95% CI	P-val	HR IP-10	95% CI	P-val
Model 1	1.47	1.22–1.77	3.0 e-5	1.41	1.16–1.73	0.0006
Model 2	1.45	1.20–1.75	9.2 e-5	1.46	1.19–1.78	0.0002
Model 3	1.4	1.15–1.70	0.0007	1.48	1.16–1.87	0.001
Model 4	1.35	1.06–1.71	0.01	1.49	1.13–2.00	0.006

Model 2 shows adjusted HRs for Age and Sex. Model 3 is a fully adjusted model for Age, Sex, GFR, Presence of Diabetes, Hb, ABI, and Fontaine Classification. Model 4 is adjusted for the same factors as model 3, with the addition of CRP.

### Discrimination and risk stratification

**Figure 3:**
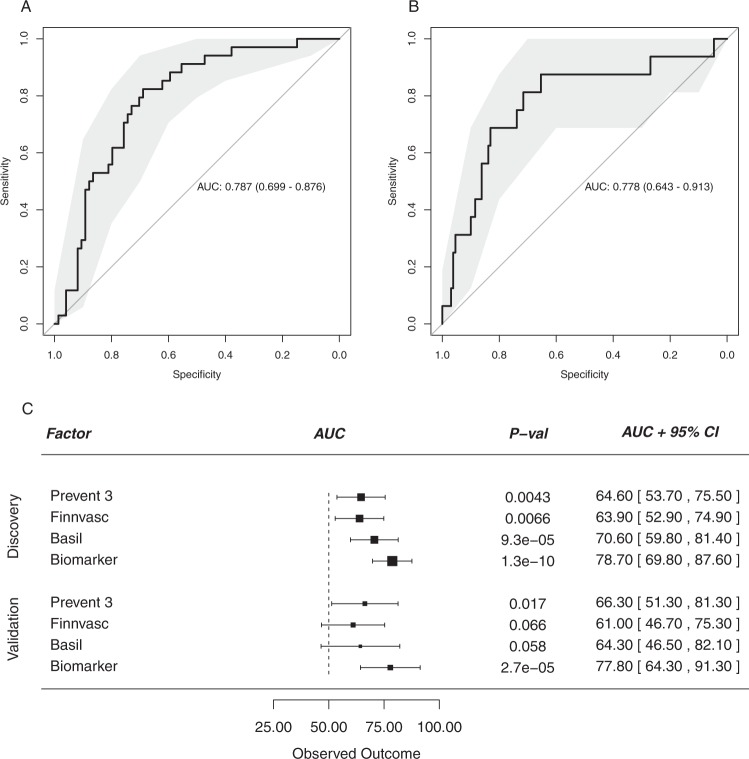
Biomarker Prediction Performance. Receiver operated characteristic (ROC) curve based on logistic regression model containing IL-6 and IP-10 (biomarker model). Shaded region indicates 95% CI based on bootstrapping (2000 iterations). (Panel A) Shows the ROC curve in the discovery cohort and (Panel B) in the validation cohort. (**C**) Areas-under curve and 95% confidence intervals of the models proposed here versus existing clinical prediction models.

In a secondary analysis, we evaluated the two biomarkers identified here for clinical risk stratification. As benchmark we used three previously validated stratification models, the Prevent 3^15^, Finnvasc^14^ and Basil^20^ and primarily investigated events at 1 year after inclusion. In the discovery cohort, 34 major events were observed at one year after inclusion. Shrunken mutually adjusted ORs for IL-6 and IP-10 were 1.75 and 2.22 respectively, with a C-statistic of 0.787 (0.70–0.88, Fig. 3A) for the combined model. Calibration plots showed that the predicted risks are close to the observed risks throughout the observed range (Supplementary Fig. 3). In the validation cohort 16 events were observed at 1 year, which is only one third of the discovery cohort. Many patients had low values of especially IL-6 and concomitant risk of events, leading to poor applicability of the model in the low predicted risk range (Supplementary Fig. 3). Nevertheless, high-risk patients were identified and discrimination performance was similar to the discovery cohort with a c-statistic of 0.778 (0.64–0.91, Fig. 3B). In both cases the biomarker-based model performed better than the previously established prediction models, which had AUCs between 0.60–0.65 (Fig. 3C).

## Discussion

In the present study we show that elevated serum levels of the inflammatory biomarkers IL-6 and IP-10 are independently associated with major clinical events in SLI patients. Patients in the highest tertiles of plasma levels of both biomarkers have more than a 12-fold increased risk of reaching a major endpoint. Correction for age, sex, renal function, DM and disease severity showed that the association is largely independent of previously identified risk factors. In addition to their mechanistic role in disease pathogenesis, we show that the biomarkers identified here are also suitable for risk stratification and allow for the creation of a prediction-model that outperforms currently available models for risk stratification in SLI.

Assessment of disease severity in patients with SLI remains difficult as physiological parameters such ABI or radiological parameters are moderately reproducible and patient-centered functional outcomes such as ambulation, pain and quality of life are subjective. Treatment decisions, including the decision to amputate the affected limb. choosing the type of revascularization, optimizing medical management or referral to specialized care settings would benefit from improved risk-stratification^21^. The biomarkers identified in this study allowed the creation of a model that predicts major clinical events with fair to good accuracy, showing an AUC of the ROC curve of 78%. This is considerably better than the existing Finnvasc^14^, Prevent 3^15^ or Basil^22^ prediction models, which perform poorly to moderately, with AUCs of ca. 60% in our study, in accordance with previous studies^21^.

It is thought that atherosclerotic disease progression in PAD is partially incited by an inflammatory response in the vascular wall^23^. Potential inflammatory triggers include traditional risk factors for cardiovascular disease, such as smoking and diabetes mellitus^24,25^. Previous studies have shown that some patients with PAD display a combination of non-traditional risk factors that are characterized by a persistent systemic inflammatory response. Such risk factors include a history of infectious disease^26^, autoimmune disease^27^, or a genetic risk^28^ with polymorphisms in genes associated with inflammation^7^. Hitherto few biomarkers have been identified that can aid in clinical decision making in patients with SLI. In other groups of patients with an elevated risk of cardiovascular events, circulating markers of systemic inflammation have been shown to be associated with future cardiovascular events and can serve as predictors for myocardial infarction or stroke^29^. In the present study, we observed that the inflammatory biomarkers IL-6, IL-8, GROα and IP-10 were predictors of major events in SLI. Within the four biomarkers identified, IL-6, IL-8 and GROα were highly correlated, most likely because they are co-regulated in the acute inflammatory response. As the primary goal of this study was risk stratification in PAD, we further pursued the specific combination of IL-6 and IP-10.

IL-6 is a soluble polypeptide that acts as one of the principle humoral regulators of the inflammatory response^30^. In particular IL-6 induces the acute phase response in the liver, leading to secretion of CRP, Serum Amyloid A and Fibrinogen^31^. Several studies have implicated IL-6 in the development of cardiovascular disease^32^, and polymorphisms in the IL-6 receptor have been associated with development of cardiovascular disease^33^. IL-6 has been shown to be an independent predictor of myocardial infarction in the Physicians’ Health study^34^, a result which has been replicated in over 25 patient cohorts, with a total of nearly 8,000 patients^35^. Literature on IL-6 in PAD has been comparatively scarce; IL-6 has been shown to be associated with decreases in ABI and the development of PAD in the Edinburgh Artery Study^11^ and a cross-sectional study has found elevated IL-6 levels in patients with severe claudication^36^. Furthermore, it was shown that patients with SLI had significantly higher IL-6 levels compared to claudicants and IL-6 levels were inversely correlated with ABI^37^. However, this is the first study to show that IL-6 predicts major clinical events in PAD, and SLI in particular.

Downstream targets of IL-6, in particular CRP have been previously associated with major events in SLI patients^38^. We found a similar association in the present study, but IL-6 proved a better predictor of major outcomes. A more direct causal involvement of IL-6 in the pathogenesis of cardiovascular disease may explain the better predictive value of IL-6^39^. Elevated levels of CRP have been linked to the presence of wound infection^38^, which has recently been taken up in the Wound, Ischemia, and foot Infection (WIfI) classification as risk factor for adverse outcomes^40^. We did not observe a statistical interaction between IL-6 levels and the presence of ulcers in the present study, and IL-6 predicted outcomes equally well in patients without ulcers.

The other independent prognostic biomarker identified in this study, IP-10, has been less extensively studied in cardiovascular disease. Pre-clinical studies have shown that IP-10 is secreted by several cell types, including monocytes, endothelial cells and fibroblasts, and can be induced by Th-17–associated cytokines^41^. It is implicated in smooth muscle proliferation and expressed in relationship to arterial damage^42^ and within atheromatous plaques^43^. It is thought that IP-10 mediates an influx of perivascular CXCR3 + macrophages^44^ and regulatory T-cells^45^ that are involved in arterial remodeling. IP-10 is mainly involved in intimal and medial hyperplasia and IP-10 knock-out mice are protected from atherogenesis^45^. Fewer studies have investigated IP-10 in human subjects. IP-10 has been shown to be elevated in patients with recurrent coronary artery disease^46,47^ and associated with poor collateral development within these patients^48^. Paradoxically, IP-10 levels are reduced in the acute phase after MI^49^. Recently, a study by Ko *et al*.^50^ identified IP-10 as a biomarker for Kawasaki disease, an inflammatory vasculitis that preferentially affects coronary arteries. These studies suggest that IP-10 plays an important role in arterial inflammation, especially of the coronary arteries and is associated with defective arteriogenesis in the heart.

Surprisingly, vascular damage markers such as sICAM-1 and sVCAM-1 did not predict major events, despite being highly elevated in comparison to healthy controls. We also observed greatly increased levels of VEGF, which similarly poorly predicted endpoints. It is conceivable that a sort of plateau in endothelial damage was reached, with compensatory angiogenesis and endothelial proliferation above which endothelial markers provide no further discriminative information. Alternatively the major events, amputation and death, that we recorded as primary outcome could be mostly brought about by macrovascular arterial disease, whereas endothelial damage is mostly related to microvascular disease.

The present study has several limitations: firstly we only recorded all-cause mortality and therefore cannot discriminate between deaths attributable to cardiovascular disease and other causes. Considering the very high rate of mortality for this age group and the high prevalence of specific cardiovascular risk factors and co-morbidity, it is likely that most deaths were due to cardiovascular events. Ideally, results should be replicated in a dedicated epidemiological cohort. Secondly, the validation cohort, comprised of the last third of the JUVENTAS cohort and a series of patients from the AtheroExpress (AE) registry is very heterogeneous as the two subgroups are fundamentally different. Whereas JUVENTAS included only no-option patients, patients were included in AE after endovascular plaque removal and were thus by definition eligible for intervention. On the whole AE patients had milder disease and lower risk of events. Despite this, the model developed in no-option SLI patients was in general still applicable. The model did not discriminate well within patients with a low risk (<5%), but it did identify these patients correctly as a group and gave accurate risk predictions for patients with a risk >5–10%. Lastly, the panel of cytokines, growth factors and endothelial damage markers under investigation was limited and designed mostly to include representative markers of larger “families” (e.g. angiogenic markers, acute inflammation, stem cell function etc.). Multiplex immunoassays can currently discriminate close to 200 markers simultaneously, which could be employed to do less biased searches^51^. The fact that the measurements were performed on cryopreserved plasma constitutes a further limitation of the study. Especially interleukins have been shown to be unstable in cryostorage at −80 °C, most notably IL-1β and IL-10^52^, which we excluded for this reason. It must also be noted that the assays used in this report have not undergone extensive validation for clinical use. While the measured values have proven consistent between the discovery and validation cohorts, the absolute values reported here must be interpreted with caution as differences between machines and centers may well occur.

The association between inflammation and major cardiovascular events in patients with SLI observed in this study appears robust. Recent studies have shown that direct therapy with interleukin 1 antagonists may prevent cardiovascular events^53^ or ameliorate the progression of chronic heart failure^54^. It is interesting to speculate whether such therapy may also be beneficial in patients with SLI, considering the high rate of events observed in the present study. It must be noted however, that the angiogenic response to ischemia is also mediated in part by IL-1^55^, making it difficult to predict how ischemic limbs will respond to interleukin antagonism. Further research should therefore proceed with caution.

In summary, we show that inflammatory biomarkers predict major outcomes in SLI. A prediction model employing two biomarkers, IL-6 and IP-10 in addition to clinical parameters predicts the occurrence of major outcomes with good accuracy. Prediction models using inflammatory biomarkers could be instrumental in improving risk-stratification in SLI patients and thus help clinical decision making and patient consultancy in a complex and high risk population.

## Supplementary information

Supplementary Information

## Data Availability

The datasets generated during and/or analyzed during the current study are available from the corresponding author on reasonable request.
