# Supplementary material for: A Pro-Inflammatory Biomarker-Profile Predicts Amputation-Free Survival in Patients with Severe Limb Ischemia
Source: Sci Rep. 2019 Jul 24;9:10740. doi: 10.1038/s41598-019-47217-1 (PMC6656730; doi:10.1038/s41598-019-47217-1)
Supplement: Supplementary file 1 — Supplementary Information [file 41598_2019_47217_MOESM1_ESM.pdf]

Supplementary Data

A Pro-Inflammatory Biomarker-Profile Predicts Amputation and Mortality in Patients with Severe Limb Ischemia.

Hendrik Gremmels, M.D., Ph.D.; Martin Teraa, M.D., Ph.D.; Saskia C.A. de Jager, Ph.D.; Gerard Pasterkamp, M.D.,Ph.D; Gert J. de Borst, M.D., Ph.D.; Marianne C. Verhaar, M.D., Ph.D.

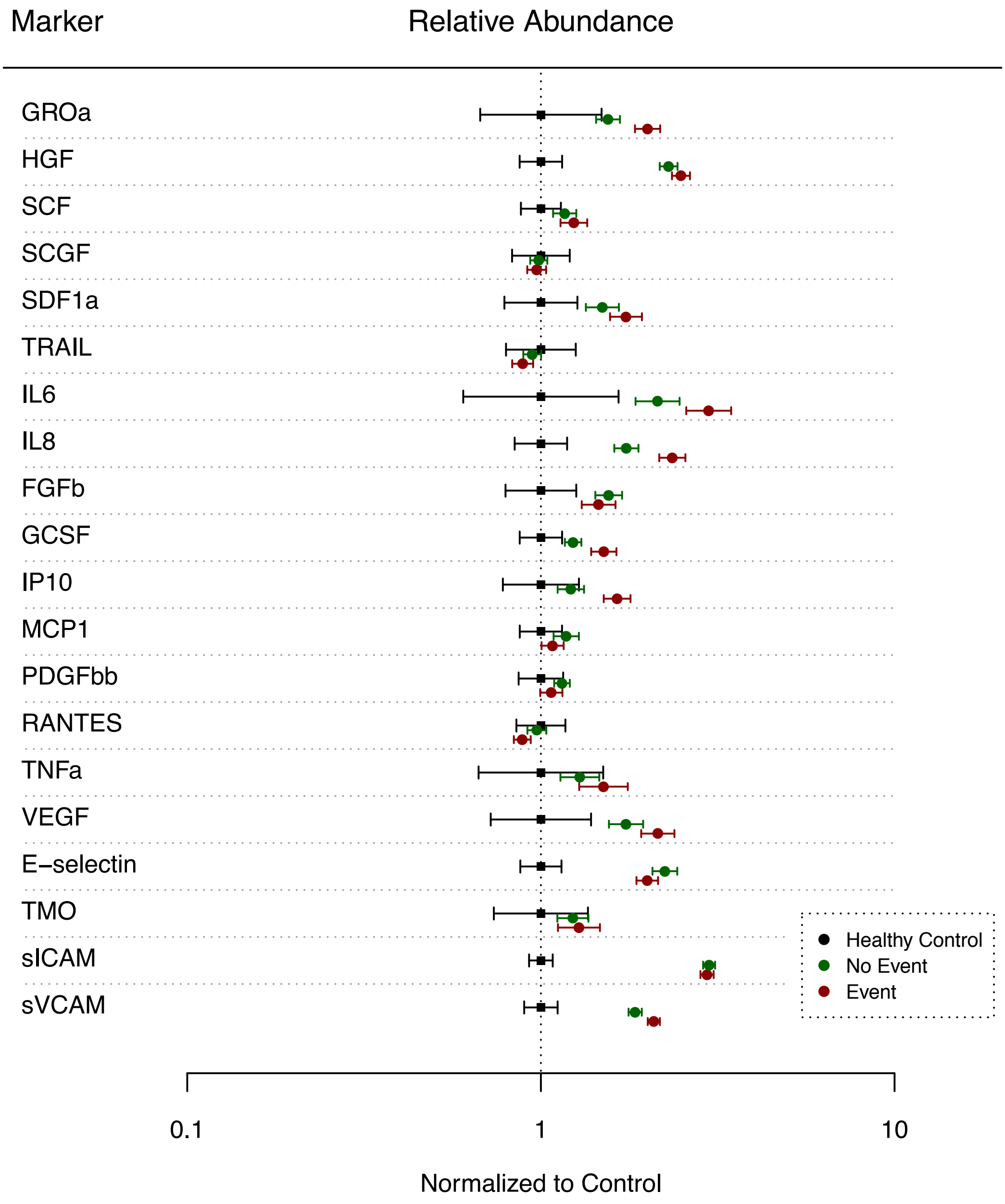

Figure 1: **Relative Abundance of Cytokines, Growth Factors or Adhesion Molecules.** Values for controls are normalized to 1 and denoted in black. Patients who underwent an event are indicated in red and patients with event-free survival are indicated in green. Error bars indicate Standard Error of the Mean (SEM)

	**Amputation**			**Death**			**AFS**		
	HR	95% CI	P-val	HR	95% CI	P-val	HR	95% CI	P-val
GROa	2.28	1.2 - 4.4	0.014	1.64	0.8 - 3.3	0.17	1.76	1.0 - 3.0	0.035
IL-6	1.92	1.4 - 2.6	5.6 e-5	1.52	1.1 - 2.1	0.006	1.58	1.2 - 2.0	0.0002
IL-8	2.66	1.6 - 4.2	6.7 e-5	1.82	1.0 - 3.2	0.037	1.923	1.3 - 2.9	0.002
IP-10	1.51	0.94 - 2.4	0.088	1.61	1.0 - 2.7	0.06	1.62	1.1 - 2.4	0.014

Table 1: **Hazard Ratios for Separate Endpoints:** This table shows the HRs in the validation cohort for Amputation, Death and the combined endpoint of Amputation Free Survival with regard to biomarker levels.

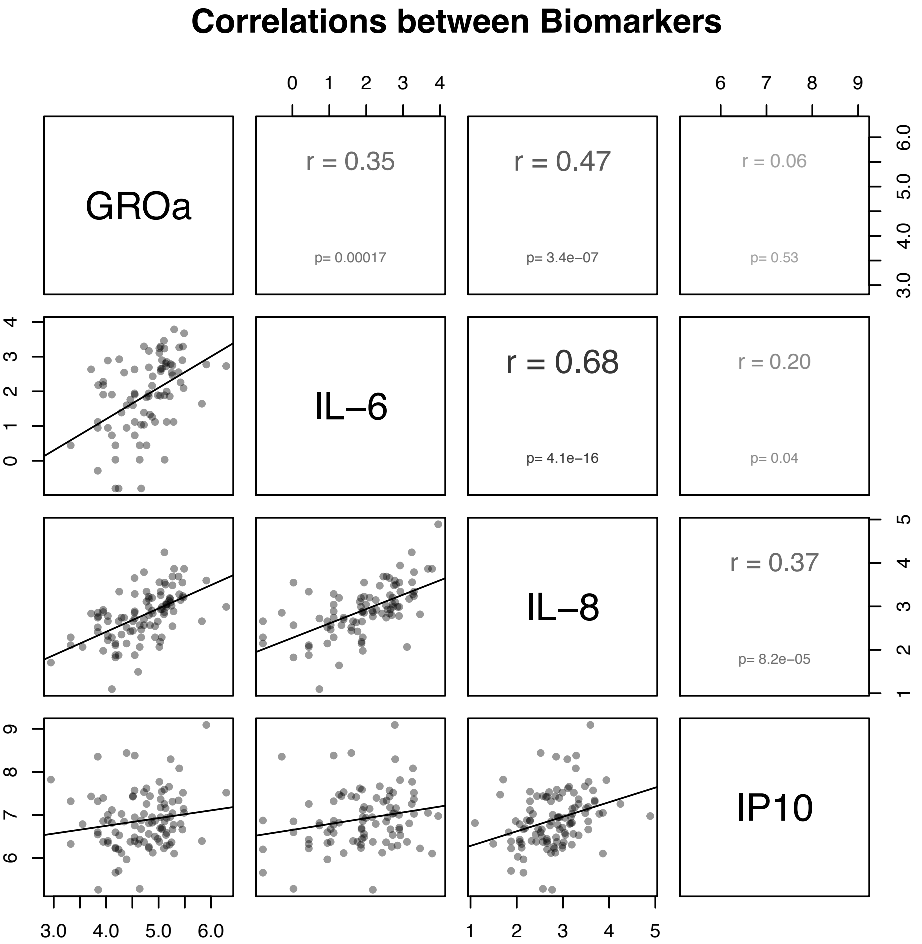

Figure 2: Correlation of Measured Cytokines

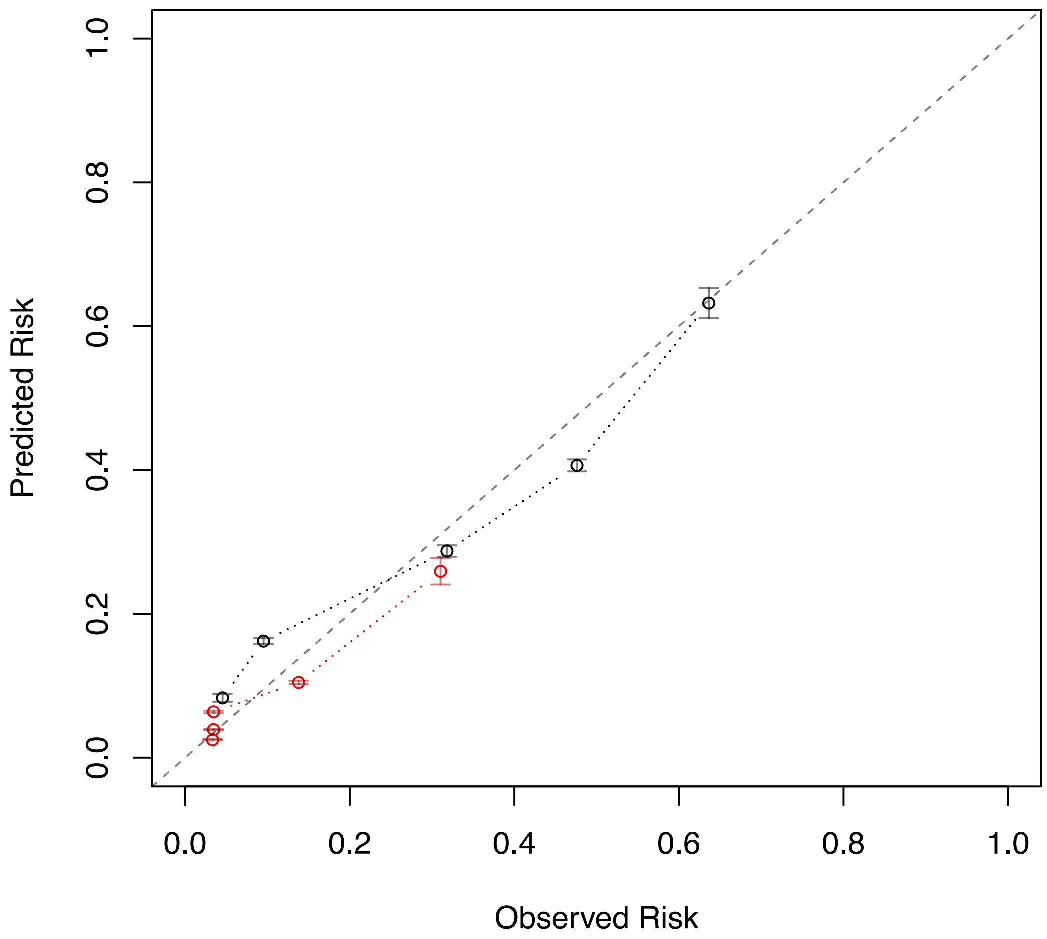

Figure 3: **Calibration of Logistic Regression Model:** Cohorts were divided into quintiles based on predicted risks and predicted risk values are compared to observed risk of major endpoints at 1 year after inclusion. The black line denotes risks in the discovery cohort (upon which the model is based), the red line risks in the validation cohort.
